# SSR molecular marker developments and genetic diversity analysis of *Zanthoxylum nitidum* (Roxb.) DC

**DOI:** 10.1038/s41598-023-48022-7

**Published:** 2023-11-26

**Authors:** Yanxia Zhu, Tao Ma, Yang Lin, Yude Peng, Yuan Huang, Jianping Jiang

**Affiliations:** 1 National Center for Traditional Chinese Medicine Inheritance and Innovation, Guangxi Botanical Garden of Medicinal Plants, Nanning, 530023 China; 2 College of Agriculture and Engineering, Guangxi Vocational University of Agriculture, Nanning, 530009 China

**Keywords:** Agricultural genetics, Genetic markers

## Abstract

*Zanthoxylum nitidum* (Roxb.) is a commonly used traditional Chinese medicine. However, the collection and protection of wild germplasm resources of *Z. nitidum* are still insufficient, and there is limited research on its genetic diversity and fingerprint. In the present study, 15 simple sequence repeat (SSR) markers were developed by genotyping based on multiplexed shotgun sequencing. The genetic diversity of 51 populations (142 individuals) of *Z. nitidum* was evaluated using these 15 SSRs. A total of 245 alleles (*Na*) were detected, with an average value of 16.333, and the average polymorphism information content was 0.756. The genetic distance among 51 populations was 0.164~1.000, with an average of 0.659. Analysis of molecular variance showed low genetic differentiation (40%) and high genetic differentiation (60%) between populations and individuals, respectively. The genetic differentiation coefficient (*Fst*) of the population was 0.338, indicating that 66.2% of the genetic variation occurred within the population, and the gene flow (*Nm*) was 0.636, demonstrating that the gene exchange between populations was low. Clustering analysis revealed that the genetic similarity coefficient was 0.30, dividing the 51 populations into 4 groups of 2, 17, 3, and 29 populations. There was no specific relationship between geographical location differences and genetic distance. The genetic diversity level of *Z. nitidum* is relatively high, and our results provide a theoretical basis for the rapid identification of* Z. nitidum* germplasm resources and variety selection.

## Introduction

*Zanthoxylum nitidum* (Roxb.) DC., a notable medicinal plant, belongs to the genus *Zanthoxylum* in the Rutaceae family, the root used as traditional medicine is named Liang-Mian-Zhen^1^. *Z. nitidum* was first recorded in "Shennong Materia Medica Classic" during the Qin and Han dynasties under the name "Manjiao"^2^. It mainly grows in Guangxi, Guangdong, and other locations in China^3^. The roots mainly contain alkaloids^4,5^, sesquiterpenoids^6^, coumarins^7^, lignans^8^, and other components that have various pharmacological activities, such as antiinflammatory^9,10^, antibacterial^11,12^, anticancer^13^ and analgesic^14^ activities. *Z. nitidum* is not only the main raw material of more than 60 famous traditional Chinese patent medicines and simple preparations, such as Sanjiu Weitai Granules, LiangMianZhen Analgesic Tablets, and Dieda Wanhua Oil, but its extracts are widely used in toothpaste, soap, shampoo and other daily personal products^15,16^.

*Zanthoxylum nitidum* can be subdivided into *Z. nitidum* var. *nitidum* and *Z. nitidum* var. *tomentosum* according to whether each section of the plant has short, rough hairs, particularly on both sides of the leaves. Furthermore, *Z. nitidum* var. *nitidum* is separated into three types based on the number of thorns on branches and leaf axes, as well as the size and thickness of leaflets^3,17^. Due to the high demand for *Z. nitidum* in the market, wild resources have been plundered recklessly for a long time, and have become increasingly scarce^17,18^. The fundamental measure to solve the resource crisis is to transform the source from wild to artificially planted. However, in the process of introducing and domesticating wild *Z. nitidum* germplasm, it is difficult to accurately distinguish resources with such similar traits using traditional morphological identification methods. Hence, there is a serious shortage of excellent germplasm basic materials for artificial cultivation, and the unclear phenotypic characteristics and genetic background of cultivated germplasm are unclear, which seriously limits the development of related industries for *Z. nitidum*.

Some of the advantages of simple sequence repeat (SSR) molecular markers are rich polymorphism, wide distribution, easy operation, and high sensitivity^19^. SSR molecular markers have been widely used in genetic diversity analysis, population structure analysis, and fingerprint construction of medicinal plants, such as for Astragali Radix^20^, *Akebia trifoliata*^21^, and *Paris polyphylla*^22^, providing new ideas for the protection of core germplasms, functional gene identification, variety identification, and molecular marker assisted breeding of medicinal plants. However, studies on the genetic diversity of *Z. nitidum* are limited, and one of the most important reasons is the lack of effective molecular markers for this species.

In this study, great efforts were made to collect wild germplasm resources of *Z. nitidum* from Guangxi and Guangdong. We detected an abundance of SSRs based on the multiplexed shotgun genotyping (MSG) data, and we developed SSR molecular markers to assess the level of genetic diversity for the collected germplasm resources. Our data will provide a scientific basis for species identification and selective breeding of *Z. nitidum.*

## Materials and methods

### Plant materials and DNA extraction

From September to October 2022, a total of 142 *Z. nitidum* var. *nitidum* and *Z. nitidum* var. *tomentosum* individuals from 51 populations in Guangdong and Guangxi were collected, including 16 germplasms from 7 artificially cultivated populations and 126 germplasms from 44 wild populations. The germplasm numbers and source information are shown in Table S1. Fresh young leaves were collected and stored at − 80 °C for DNA extraction. Total genomic DNA was extracted from the frozen leaves following the instructions of Magnetic Bead Method using a Plant Genomic Extraction Kit (NanoMagBio, Wuhan, China). The concentration and purity of DNA were determined by using an ultramicro spectrophotometer (NanoDrop ONE, Thermo Fisher, USA).

### MSG library construction and sequencing

Three germplasms with significant phenotypic differences (GX0826-1, bl0915-1, 3-1) were selected for constructing the library. We prepared the sequencing library using the MSG method proposed by Andolfatto et al.^23^. We purified the libraries and selected DNA fragments in the 400 bp size range using AMPure XP beads (Beckman Coulter, Inc., USA), and then amplified them using PCR for 14 cycles. The Illumina NovaSeq platform (Illumina, Inc., San Diego, USA) was used to obtain raw sequence data. The original data were filtered using the sliding window analysis method of fastp (v0.20.0)^24^, and the sequences were integrated using FLASH (v1.2.11)^25^ to obtain high-quality data. The sequencing data have been deposited in China National GeneBank (CNGB) with project accession number CNP0004226 (https://db.cngb.org/search/project/CNP0004226/).

### SSR primer pair design

The Microsatellite Identification Tool (MISA) (http://pgrc.ipk-gatersleben.de/misa/) was used to search for SSR loci in all high-quality sequences. Cd-hit software^26^ was used to cluster the sequences. The Perl program was used to analyze the clustering results and evaluate polymorphisms^27^. Finally, PCR primer pairs flanking the SSR repeats were designed using primer 3 (v2.3.6)^28^.

### SSR analysis and screening

SSRs were classified based on the length of the SSR motifs in their sequences. Different repeats of the SSR motifs were used to analyze the characteristics of the SSRs. We randomly selected 192 pairs of primers, and preliminarily screened 10 germplasms with significant phenotypic differences (GX0826-1, bl0915-1, 3-1, te0920-1, jx0928-1, pg1001-1, tl1004-1, qb1007-1-1, nv-1, py1-1). PCR reaction was performed using a Veriti 384 PCR instrument(Veriti 384, AppliedBiosystem,USA). The PCR reaction system was as follows: template DNA 1 μL, forward and reverse primers are both 10 pmol/μL (Shanghai, Shanghai), 0.5 μL each, 5 μL of MIX enzyme (2 × Taq PCR Master Mix, GeneTech, USA) supplemented with 3 μL ddH_2_O (total 10 μL). The reaction conditions were as follows: predenaturation at 95 °C for 5 min, 25 cycles of denaturation at 95 °C for 30 s, gradient annealing at 62~52 °C for 30 s, extension at 72 °C for 30 s, followed by extension at 72 °C for 20 min. Polymorphism of each successfully amplified marker was evaluated by GeneMarker software.

### Fluorescence capillary electrophoresis detection

Fluorescent primers were obtained from Wuhan Tianyi Huiyuan Biotechnology Co., Ltd. (Wuhan, China), and the fluorescent dyes used were FAM, HEX, and TAMRA. One microliter of fluorescent PCR product, 0.5 μL of GeneScan™500 LIZ, and 8.5 μL of Hi-Di™ formamide were added to the upper plate, centrifuged, denatured (95 °C for 5 min), and cooled. Finally, the samples were analyzed using an ABI3731XL sequence analyzer (AppliedBiosystem, USA).

### Genetic diversity analysis

GenAlEx (v6.501) software was used to calculate genetic diversity indicators^29^, including the number of observed alleles (*Na*), effective alleles (*Ne*), observed heterozygosity (*Ho*), expected heterozygosity (*He*), fixation index (*F*), Shannon’s information index (*I*), genetic differentiation coefficient (*Fst*), gene flow (*Nm*) and analysis of molecular variance (*AMOVA*). Linkage Disequilibrium between the SSR loci was carried out using the SHEsis plus^30^. The genetic distance among populations was calculated using Powermarker (V3.25)^31^. The genetic structure of the germplasm was analyzed using STRUCTURE software (v2.3.4)^32^, and cluster analysis between populations was based on unweighted pair group with arithmetic average (UPGMA).

### Plant collecting permit declaration

The plant materials used in this article did not involve disputes. We hereby declare that all of the plant materials (*Z. nitidum* var. *nitidum* and *Z. nitidum* var. *tomentosum*) were collected in compliance with institutional, national, and international guidelines and legislation. The plant material in our collection is preserved in the Germplasm Repository of Guangxi Botanical Garden of Medicinal Plants, Guangxi Province, China. The voucher ID for each sample is shown in Table S1. The formal identification of these plant materials was performed by Prof. Yudeng Peng (Guangxi Botanical Garden of Medicinal Plants, China).

## Result analysis

### SSR quantity analysis

MSG data of the three samples (GX0826-1, bl0915-1, and 3-1) were 8.63 Gb, 6.66 Gb, and 9.35 Gb, respectively. After filtering, high-quality data were generated, which were 1.30 Gb, 0.93 Gb, and 1.20 Gb, respectively. Furthermore, 2,903,693 pairs of read pairs were merged from a total of 12,014,757 pairs. Using MISA software, 261,267 SSR loci were found, distributed among 227,023 unigenes. The frequency of SSR occurrence (the proportion of sequences containing SSR loci to all sequences) was 7.82%. The average distance (total sequence length divided by the total number of SSRs) was 2.73 kb. Among them, there were 30,512 unigenes containing more than one SSR locus, and 29,314 SSRs were present in composite form (Table 1).

**Table 1:** SSR search results based on MSG data.

Item	Number
Number of sequences analyzed	2,903,693
Length of sequences/bp	713,743,318
Number of SSR	261,267
Number of sequences with SSRs	227,023
Number of sequences with more than one SSRs	30,512
Number of SSRs present in compound formation	29,314
Occurrence frequency of SSR / %	7.82
Average distance/kb	2.73

### SSR polymorphisms

SSRs with mono, di-, tri-, tetra-, penta-, and hexanucleotide repeat units were identified based on MSG data. Among the SSRs, mononucleotide (207,994, 79.61%) was the most abundant repeat type, followed by dinucleotide loci (32,332, 12.38%), trinucleotide loci (17,637, 6.75%), tetranucleotide loci (2265, 0.87%), hexanucleotide loci (525, 0.20%), and pentanucleotide loci (514, 0.20%). Moreover, the three types of repetitive motifs with the highest number were A/T (207,789, 79.53%), AT/AT (19,881, 7.61%), and AAT/ATT (8245, 3.16%) (Table 2).

**Table 2:** Characterization of SSR loci.

Repeat type	Repeat motif	Number	Percentage
Mononucleotide	A/T	207,789	79.53
	G/C	205	0.08
	Total	207,994	79.61
Dinucleotide	AC/GT	4879	1.87
	AG/CT	7503	2.87
	AT/AT	19,881	7.61
	CG/CG	69	0.03
	Total	32,332	12.38
Trinucleotide	AAC/GTT	1097	0.42
	AAG/CTT	3280	1.26
	AAT/ATT	8245	3.16
	ACC/GGT	858	0.33
	ACG/CGT	271	0.10
	ACT/AGT	97	0.04
	AGC/CTG	1156	0.44
	AGG/CCT	502	0.19
	ATC/ATG	1667	0.64
	CCG/CGG	464	0.18
	Total	17,637	6.75
Tetranucleotide	Total	2265	0.87
Pentanucleotide	Total	514	0.20
Hexanucleotide	Total	525	0.20

### SSR marker development

On the basis of the identified SSR loci, 768 primer pairs were designed (Table S2). Among the 192 randomly selected SSR markers (Table S3) screened using 10 selected populations, 39 SSR markers with polymorphisms were preliminarily screened, with a polymorphism rate of 20.31%. Then, 15 randomly selected SSR markers were used for genetic diversity analysis of 51* Z. nitidum* populations (Table 3).

**Table 3:** List of 15 SSR markers used in the present study.

locus	Repeating motif	Forward primer	Reverse primer	Expected product size / bp	Fluorescent labeling
ZYX006	(GT)11	GGGGAAACCATTGAGCATAA	ACCTCTTGGAATCGGTGACA	166	FAM (blue)
ZYX149	(AG)6	GGTGGTGCCAGCTGAATTAT	CCGATAACAAGAGCATGGGT	211	FAM (blue)
ZYX148	(CAG)6	TGAACACACCGTCTCCTCAG	GACTGCCTGAACTGTTGCTG	133	FAM (blue)
ZYX119	(TG)7	TGCAACAAGACTAATGCACAAA	TGAGTATGATTGCAGGTGCC	190	FAM (blue)
ZYX137	(AG)8	TCGTGATTGCGAAGATTAGG	ATGCGCATCTTCTTCAGCTT	243	FAM (blue)
ZYX031	(ATA)6	CCATAAACTTTCCTCTTTCGATG	CCAGCAAAAGCTCCAGAATC	134	FAM (blue)
ZYX163	(ATT)5	CGGAGGCTATTCTGCATTTC	CGAATCGAGACCGTTCATTT	178	FAM (blue)
ZYX050	(AT)7	AAATCCGAGATACCTTTTAAAGCA	ACTCAACTGCAGAATCGGCT	155	FAM (blue)
ZYX191	(AG)10	AGACACACAAGCACACGAGC	CTAAGCGACCGACATTCAGG	142	FAM (blue)
ZYX092	(ATA)8	TGAAGCAACTGCTAGCAACG	CTGGTCCTCCCTTCCTCTCT	213	FAM (blue)
ZYX004	(GT)9	TCCCTTGCAATTCCCAATAC	CTCTTCTTGCTGCCGCTACT	153	FAM (blue)
ZYX182	(CTT)7	AAGCAACCAAAAGCGTCACT	CCCGATTATGGGGTAGGATT	193	FAM (blue)
ZYX022	(ATT)18	TTGGAAGCCATCTGACTGTG	CGGGATTGGAAGAGTACTGG	212	FAM (blue)
ZYX171	(TA)7	TGCAGCAAACCGTTTACAAG	GAGCGAGTCTGGAGAAGCAG	129	FAM (blue)
ZYX056	(AG)9	GCTCAAAACAAGAGCACGAA	TCCACTCTGCTATTTCTGCTAGG	230	FAM (blue)

### SSR marker detection

**Figure 1:**
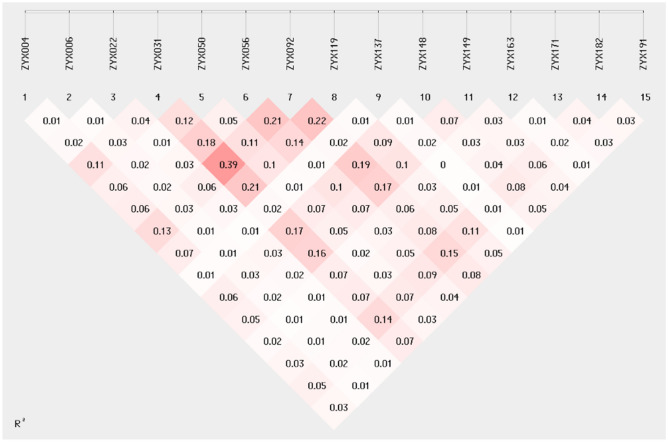
Linkage disequilibrium distribution pattern based on R^2^ values between SSR loci. The color of the box ranges from white to red, representing the level of linkage disequilibrium from low to high.

A total of 245 alleles (*Na*) from 15 developed SSR markers were amplified from the 51 populations (142 individuals). The total effective alleles (*Ne*) were 85.828, with an average of 5.722, and the proportion of effective alleles was 35.03%. Shannon’s information index (*I*) ranged from 1.313 to 2.994, with an average value of 1.984. The polymorphism information content (*PIC*) ranged from 0.531 to 0.922, with an average of 0.756. All loci were not conforming to the Hardy–Weinberg equilibrium (Table 4). The linkage disequilibrium distribution pattern based on R^2^ values between SSR loci is shown in Fig. 1. R^2^ ranged between 0.01 and 0.39, indicating a relatively low degree of linkage. These results show that the 15 loci those were chosen have a high degree of polymorphism and can be utilized for further research on* Z. nitidum*'s genetic diversity.

**Table 4:** Genetic diversity parameters of 15 SSR markers.

locus	*Na*	*Ne*	*I*	*Ho*	*He*	*F*	*PIC*	*P* value	Significance
ZYX004	9	5.567	1.896	0.440	0.820	0.464	0.798	0.000	***
ZYX006	12	6.463	2.007	0.681	0.845	0.195	0.826	0.000	***
ZYX022	32	9.876	2.902	0.676	0.899	0.248	0.894	0.000	***
ZYX031	10	2.567	1.36	0.270	0.610	0.559	0.580	0.000	***
ZYX050	15	2.728	1.510	0.359	0.633	0.433	0.604	0.000	***
ZYX056	11	3.473	1.668	0.411	0.712	0.422	0.686	0.000	***
ZYX092	13	2.271	1.313	0.114	0.560	0.796	0.531	0.000	***
ZYX119	11	3.533	1.669	0.279	0.717	0.611	0.691	0.000	***
ZYX137	33	13.464	2.994	0.660	0.926	0.288	0.922	0.000	***
ZYX148	9	4.760	1.704	0.493	0.790	0.376	0.759	0.000	***
ZYX149	14	4.993	1.925	0.578	0.800	0.278	0.775	0.000	***
ZYX163	37	6.226	2.572	0.546	0.839	0.349	0.829	0.000	***
ZYX171	12	6.120	1.996	0.657	0.837	0.215	0.817	0.000	***
ZYX182	10	4.458	1.793	0.411	0.776	0.470	0.751	0.000	***
ZYX191	17	9.329	2.457	0.637	0.893	0.286	0.884	0.000	***
Mean	16	5.722	1.984	0.481	0.777	0.399	0.756		
St Dev	9	3.104	0.523	0.174	0.110	0.165			

The P-value is the testing result of the Hardy–Weinberg equilibrium, and the smaller the p-value, the loci that do not conform to expectations of Hardy–Weinberg equilibrium.

### Population genetic diversity

Statistical analysis showed that the maximum genetic distance among 51 populations was 1.000, the minimum was 0.164, and the average was 0.659 (Table S4). The results obtained for population-level indices of genetic diversity in each population are shown in Table 5. The average values of *Na*, *Ne*, *I*, *Ho*, *He* and *F* at the population level were 2.220, 1.964, 0.619, 0.478, 0.380, and − 0.268, respectively. Taken together, these results indicate that POP15 and POP1 exhibit the highest and lowest levels of genetic diversity, respectively.

**Table 5:** Genetic diversity of 51 populations.

Pop	Na	Ne	I	Ho	He	F
POP1	1.267	1.267	0.185	0.267	0.133	− 1.000
POP2	2.600	2.134	0.736	0.622	0.430	− 0.441
POP3	2.933	2.482	0.881	0.578	0.504	− 0.173
POP4	2.267	1.928	0.635	0.356	0.389	0.060
POP5	2.067	1.796	0.567	0.422	0.359	− 0.170
POP6	2.267	2.071	0.704	0.467	0.450	− 0.036
POP7	1.467	1.364	0.274	0.233	0.183	− 0.267
POP8	2.200	1.767	0.590	0.389	0.362	− 0.073
POP9	2.200	2.058	0.629	0.600	0.383	− 0.573
POP11	2.333	2.018	0.643	0.333	0.381	0.079
POP12	2.267	1.872	0.621	0.489	0.378	− 0.274
POP13	1.733	1.640	0.430	0.467	0.283	− 0.667
POP14	1.667	1.503	0.390	0.378	0.263	− 0.393
POP15	3.267	2.927	1.043	0.733	0.600	− 0.239
POP16	2.133	1.940	0.628	0.489	0.399	− 0.237
POP17	2.333	1.968	0.600	0.400	0.342	− 0.168
POP18	1.933	1.769	0.514	0.333	0.325	− 0.007
POP19	1.800	1.607	0.415	0.356	0.259	− 0.320
POP20	2.933	2.527	0.907	0.444	0.522	0.106
POP21	2.800	2.393	0.855	0.533	0.500	− 0.032
POP22	2.667	2.238	0.792	0.467	0.467	0.026
POP23	2.600	2.398	0.827	0.711	0.504	− 0.437
POP24	1.867	1.633	0.464	0.467	0.296	− 0.549
POP25	1.400	1.400	0.277	0.400	0.200	− 1.000
POP26	1.467	1.467	0.323	0.467	0.233	− 1.000
POP27	3.000	2.455	0.880	0.578	0.496	− 0.147
POP28	2.133	1.941	0.553	0.489	0.326	− 0.508
POP29	1.533	1.520	0.366	0.511	0.263	− 0.938
POP30	1.733	1.570	0.363	0.267	0.219	− 0.244
POP32	1.733	1.692	0.492	0.689	0.352	− 0.927
POP33	2.333	1.946	0.677	0.622	0.419	− 0.447
POP34	1.667	1.626	0.446	0.622	0.319	− 0.920
POP35	1.667	1.544	0.406	0.467	0.278	− 0.648
POP36	2.400	2.056	0.683	0.467	0.411	− 0.114
POP37	1.733	1.733	0.508	0.733	0.367	− 1.000
POP38	2.533	2.172	0.704	0.433	0.403	− 0.062
POP39	2.400	2.002	0.658	0.422	0.389	− 0.106
POP40	1.600	1.559	0.400	0.556	0.285	− 0.911
POP41	2.533	2.167	0.755	0.300	0.454	0.428
POP42	2.533	2.020	0.703	0.444	0.411	− 0.133
POP43	2.200	1.899	0.607	0.556	0.374	− 0.472
POP44	2.867	2.600	0.970	0.533	0.583	0.105
POP45	2.467	2.114	0.739	0.456	0.453	− 0.014
POP46	2.067	1.942	0.621	0.333	0.408	0.189
POP47	2.867	2.485	0.843	0.511	0.478	− 0.072
POP48	2.533	2.256	0.715	0.467	0.419	− 0.087
POP49	2.133	1.794	0.554	0.467	0.337	− 0.371
POP50	2.400	2.187	0.713	0.533	0.425	− 0.261
POP51	2.933	2.517	0.886	0.556	0.507	− 0.090
POP52	2.267	2.021	0.619	0.422	0.367	− 0.083
POP53	2.467	2.160	0.774	0.567	0.475	− 0.210
Mean	2.220	1.964	0.619	0.478	0.380	− 0.268
Max	3.267	2.927	1.043	0.733	0.600	0.428
Min	1.267	1.267	0.185	0.233	0.133	− 1.000

### Genetic differentiation

According to AMOVA, 40% of the total genetic variation originated from variability among the populations, whereas 60% was attributed to within-individual differences (Table 6). *F*-statistical analysis revealed that the genetic differentiation coefficient (*Fst*) of the population was 0.338, and the gene flow (*Nm*) was 0.636 (Table S5).

**Table 6:** Analysis of the molecular variance (AMOVA) of the 142 individuals.

Source of variation	df	SS	MS	Est. var	Variation percentage (%)
Among populations	50	835.553	16.711	2.355	40
Within individuals	142	503.500	3.546	3.546	60
Total	283	1666.803		5.929	100

*df* degree of freedom, *SS* Total variance, *MS* Mean square deviation, *Est. Var.* Estimated difference value.

### Phylogeny tree

**Figure 2:**
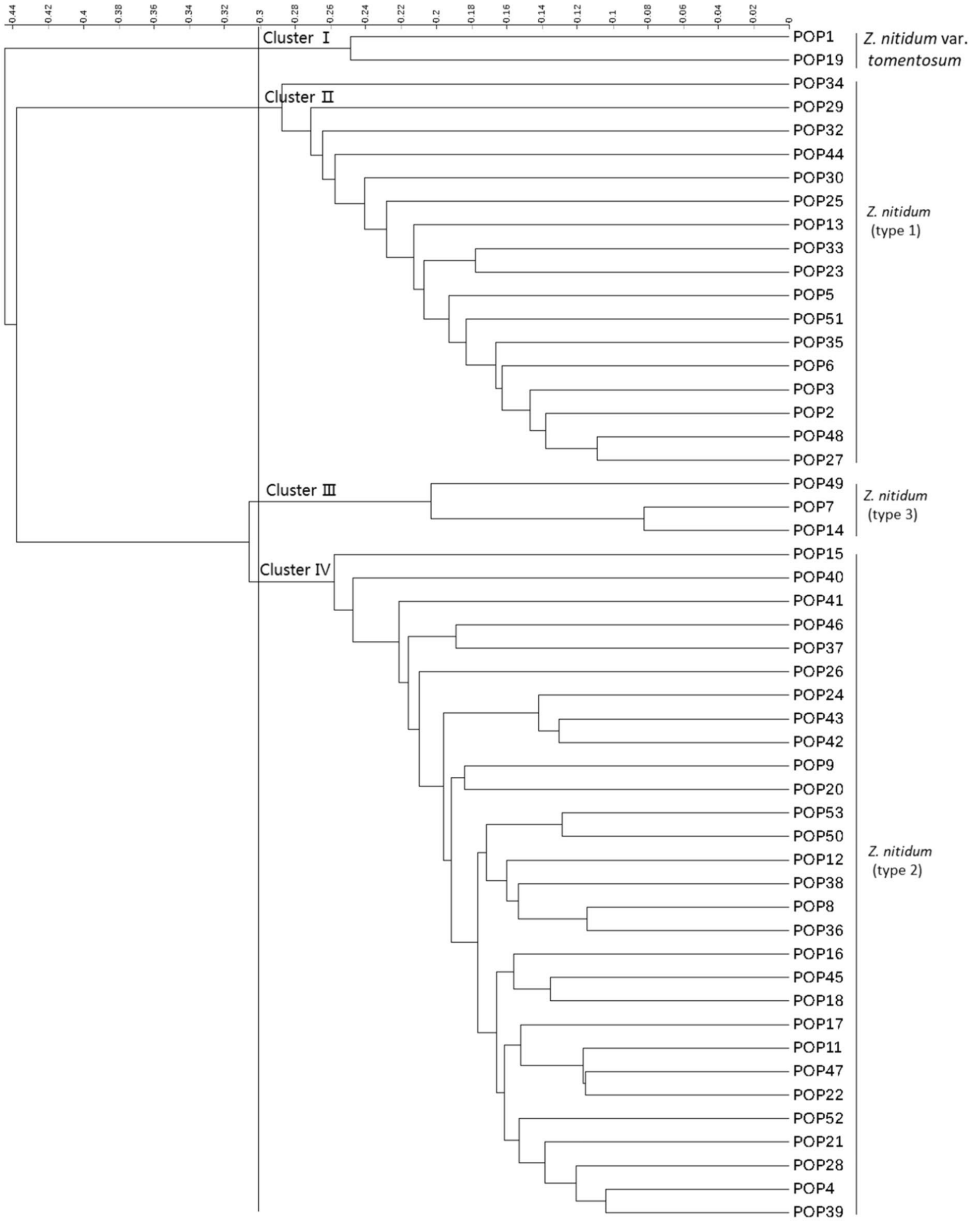
Phylogeny tree of 51 populations.

According to Nei's genetic similarity coefficient, the UPGMA method was used to construct a phylogenetic tree. The genetic similarity coefficient variation of 51 populations ranged from 0 to 0.44, and when the similarity coefficient was 0.30, they could be clustered into 4 clusters (Fig. 2). Cluster I contained 2 populations, both of which have been identified as *Z. nitidum* var. *tomentosum*. Cluster II contained 17 populations, all of which have been identified as type 1 of *Z. nitidum* var. *nitidum*. Cluster III included 3 populations from Guangdong, all of which have been identified as type 3 of *Z. nitidum* var. *nitidum*. Cluster IV contained 29 populations, including all 7 cultivated populations, all of which have been identified as type 2 of *Z. nitidum* var. *nitidum*.

## Discussion

SSRs, as important molecular markers, have been widely used in genetic diversity evaluation, genetic map, construction, and finger mapping in medicinal plants, such as for the medicinal plants *Aristolochia delavayi*^33^, *Andrographis paniculata*^34^, and *Paris polyphylla*^35^. However, genetic research on *Z. nitidum* has been hampered due to limited genetic information. MSGs were used for high-throughput discovery of SSRs^23^. It was confirmed that MSG is similar in essence to restriction site-associated DNA (RAD) sequencing^36^ and whole-genome resequencing (WGD)^37^, and it is more effective and flexible for detecting SSRs. In this study, 261,267 SSR loci by MSG were discovered.

The number of *Na*, *Ne*, *Ho*, *He*, *F*, *I*, and *PIC* of primers are important indicators for measuring polymorphism of SSR markers. When PIC > 0.5, it indicates that the primer has a high degree of polymorphism^38^. In this study, we designed 768 SSRs based on MSG data, and screened out specific markers of *Z. nitidum* form them. To evaluate the effectiveness of the designed SSR primers, we randomly selected 192 SSRs and conducted two rounds of screening based on 10 germplasms with significant phenotypic differences. Twenty-six SSRs were found to be effective. Fifteen SSRs were selected from 26 SSRs for genetic diversity analysis on 142 individuals. The *PIC* of these 15 SSRs ranged from 0.531 to 0.922, with an average of 0.756. These 15 SSRs have high polymorphism and can effectively reveal the genetic diversity of *Z. nitidum*. This is the first time SSR markers have been used for genetic diversity analysis of *Z. nitidum* populations; at the same time, our results confirm that SSR markers have the advantages of a large number of loci and high identification efficiency.

Among the indicators of population genetic diversity, expected heterozygosity (*He*)^39^ and Shannon’s information index (*I*)^40^ are important. The larger their values are, the richer the genetic diversity of the population is. This study found that the *He* of these 51 populations ranged from 0.133 to 0.600, with an average of 0.380; the *I* ranged from 0.185 to 1.043, with an average of 0.619. These data indicate that the 51 populations have rich genetic diversity. In general, *Fst* > 0.25 indicates a high level of genetic differentiation among populations^41^; *Nm* < 1 indicates that the genetic differentiation among populations is caused by migration or genetic drift^42^. In this study, the *Fst* of 51 populations was 0.338, and the *Nm* was 0.636, which indicated that the genetic differentiation among populations of *Z. nitidum* is at a high level. Additionally, the genetic differentiation between populations was caused by migration or genetic drift, which is consistent with the conclusions reached by previous researchers using ISSR molecular markers^43^, and similar to the results of genetic differentiation studies of other *Zanthoxylum* plants^44,45^.

Conducting variety identification by combining molecular marker technology with phenotypic analysis is reliable and convenient^46,47^. In our study, we constructed a UPGMA clustering tree using the 15 SSRs based on Nei's genetic similarity coefficient. Fifty-one populations could be clearly clustered into 4 clusters. Among them, the first cluster was identified as *Z. nitidum* var. *tomentosum*, indicating distant genetic relationship between *Z. nitidum* var. *tomentosum* and the rest of the germplasm with relatively clear separation. The second cluster was identified as *Z. nitidum* var. *nitidum* (type 1), indicating a distant genetic relationship between type 1 and the other two types. The third cluster was identified as *Z. nitidum* var. *nitidum* (type 3), all of which originated from Guangdong, showing obvious regional characteristics. The cluster was identified as *Z. nitidum* var. *nitidum* (type 2), with the closest genetic relationship to type 3, and included 7 artificially cultivated populations, indicating a decrease in genetic diversity after transitioning from wild to domestic. The conclusion of the genetic relationship between the four types of* Z. nitidum* through cluster analysis is consistent with traditional morphological identification methods and proves the effectiveness of the SSR molecular markers selected in this study for identifying *Z. nitidum* germplasm.

## Conclusion

In this study, MSG data were used to develop SSRs, and 261,267 SSRs were detected. Fifteen SSRs were powerful molecular markers for genetic diversity analysis and variety identification in* Z. nitidum*. Our data will be useful for germplasm identification, genetic improvement, and variety selection.

### Supplementary Information

Supplementary Table S1.

Supplementary Table S2.

Supplementary Table S3.

Supplementary Table S4.

Supplementary Table S5.

Supplementary Table S6.

## Data Availability

The data that support the findings of this study are available in the supplementary material of this article.
